# Identifying Cell Types from Spatially Referenced Single-Cell Expression Datasets

**DOI:** 10.1371/journal.pcbi.1003824

**Published:** 2014-09-25

**Authors:** Jean-Baptiste Pettit, Raju Tomer, Kaia Achim, Sylvia Richardson, Lamiae Azizi, John Marioni

**Affiliations:** 1 European Bioinformatics Institute-European Molecular Biology Laboratory (EMBL-EBI), Cambridge, United Kingdom; 2 Developmental Biology Unit, European Molecular Biology Laboratory (EMBL), Heidelberg, Germany; 3 MRC Biostatistics Unit (MRC BSU), Cambridge Institute of Public Health, Cambridge, United Kingdom; University of Toronto, Canada

## Abstract

Complex tissues, such as the brain, are composed of multiple different cell types, each of which have distinct and important roles, for example in neural function. Moreover, it has recently been appreciated that the cells that make up these sub-cell types themselves harbour significant cell-to-cell heterogeneity, in particular at the level of gene expression. The ability to study this heterogeneity has been revolutionised by advances in experimental technology, such as Wholemount in Situ Hybridizations (WiSH) and single-cell RNA-sequencing. Consequently, it is now possible to study gene expression levels in thousands of cells from the same tissue type. After generating such data one of the key goals is to cluster the cells into groups that correspond to both known and putatively novel cell types. Whilst many clustering algorithms exist, they are typically unable to incorporate information about the spatial dependence between cells within the tissue under study. When such information exists it provides important insights that should be directly included in the clustering scheme. To this end we have developed a clustering method that uses a Hidden Markov Random Field (HMRF) model to exploit both quantitative measures of expression and spatial information. To accurately reflect the underlying biology, we extend current HMRF approaches by allowing the degree of spatial coherency to differ between clusters. We demonstrate the utility of our method using simulated data before applying it to cluster single cell gene expression data generated by applying WiSH to study expression patterns in the brain of the marine annelid *Platynereis dumereilii*. Our approach allows known cell types to be identified as well as revealing new, previously unexplored cell types within the brain of this important model system.

## Author Summary

Tissues within complex multi-cellular organisms have historically been defined in terms of their anatomy and function. More recently, experimental approaches have shown that different tissues express distinct batteries of genes, thus providing an additional metric for characterising them. These experiments have been performed at the whole tissue level, with gene expression measurements being "averaged" over millions of cells within a tissue. However, it is becoming apparent that even within putatively homogeneous tissues there exists significant variation in gene expression levels between cells, suggesting that additional cell subtypes, defined by distinct expression profiles, might be obscured by "bulk" experimental approaches. Herein, we develop a computational approach, based upon Markov Random Field models, for clustering cells into cell types by exploiting their gene expression profiles and location within the tissue under study. We demonstrate the efficacy of our approach using simulations, before applying it to identify known and putatively novel cell types within the brain of the ragworm, *Platynereis dumerilii*, an important model for understanding how the Bilaterian brain evolved.

## Introduction

Complex organisms are heterogeneous at several levels. For example, one can divide the body into functional organs: the skin, the brain, the liver and so on. This anatomical and functional classification implies that distinct organs are composed of different cell types. Interestingly, these functional building blocks are also not composed of homogeneous cell types. Indeed, they are composed of several tissues that together make up a complex organ. For example, the skin of mammals can be described as the superposition of the Epidermis, Dermis and Hypodermis [1]. However, even with this more precise description, each of these tissues will be heterogeneous. For instance in the Dermis, the cells making up the sweat glands will not be the same as the cells in the hair follicles. Additionally, this heterogeneity does not stop at this sub-sub classification: heterogeneity is still present and, with fine enough measurements methods, this remains true to the single cell level [2].

When reducing the scale of study, the classification of cells into distinct groups ceases to be anatomical. Instead, molecular biology has allowed scientists to define molecular characteristics that distinguish individual cells. The most widely used characteristic is mRNA expression, and gene expression signatures are now commonly used to define cell types [3], [4]. Conceptually, if a set of cells have similar expression profiles, this information can be used to gather these cells into a specific cell type; we focus on this, molecular, definition of a cell type in the remainder of this manuscript.

To do this, gene expression measurements at the single cell level within the tissues under study are necessary. Recent technological developments have facilitated this shift from tissue to single cell resolution: in-situ hybridization [5] in a few organisms including *P. dumerilii* and single cell RNA sequencing assays [6] are amongst a number of methods that allow gene expression to be measured at the single cell level [7]. Given this, one key challenge is to develop computational methods that use the expression data to cluster single cells into robust groups, which can then be examined to determine their likely functional roles.

Many popular clustering methods (e.g., hierarchical clustering, k-means and independent mixture models) exist and can be applied to address this problem [8]–[10]. However, these methods fail to take into account the spatial location of each cell within the tissue under study — when such information is available [3], [11], [12], it is extremely useful and should be incorporated into the downstream analysis. Specifically, we can hypothesise that cells that are close together are more likely to belong to the same cell type. In other words, if a cell has a "slightly" more "similar" expression profile to a typical cell in cell type *b* than in cell type *a* but all the surrounding cells have been classified as belonging to cell type *a* it seems sensible to assign this cell to cell type *a*. However, it is also important to note that cell migration, which takes place during the development of complex tissues, can lead to isolated cells with very different expression profiles than their neighbours, which also needs to be accounted for.

**Figure 1:**
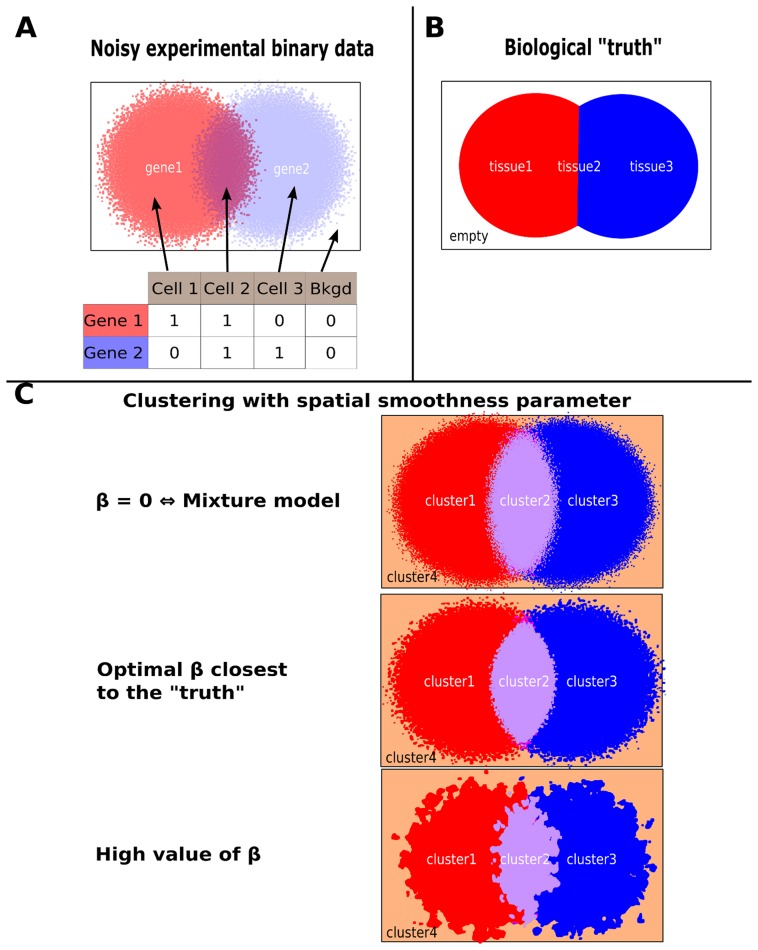
Schematic representation of the influence of spatial coherence when clustering noisy data. Panel A shows an example of noisy data for the expression of two genes as well as the resulting binarised table of expression for four cells in the assay. Panel B shows the reference represented by 3 spatially coherent cell types inside an empty area. Panel C shows the influence of the spatial smoothness/coherency parameter on the clustering results.

To address these problems and, in particular, to utilise both the spatial and the quantitative information, we extended a graph theoretical approach developed for image segmentation to reconstruct noisy or blurred images [13], a method that finds its roots in the field of statistical mechanics as the Ising model [14] and its generalization, the Potts model [15]. The core concept of this method (Figure 1) is to estimate the parameters of a Markov Random Field based model using mean-field approximations to estimate intractable values as described in [16]. We use an Expectation-Maximization (EM) procedure to maximize the parameters as described in [13], [16]. To the best of our knowledge, such methods have not previously been applied to 3-Dimensional gene expression data. Additionally, from a theoretical perspective, we extended current models by allowing the degree of spatial cohesion per cluster to differ, thus allowing for the possibility that some cell types are more spatially coherent than others. After validating our approach using simulated data, we demonstrated its utility by applying it to data generated using methods described in Tomer et al. [3] who were interested in studying the ancestral bilaterian brain.

## Results

### Motivating data: Single cell in-situ hybridization in *Platynereis dumerilii*

Tomer et al. [3] used Wholemount in Situ Hybridisation (WiSH) to study the spatial expression pattern of a subset of genes, at single cell resolution, in the brain of the marine annelid *Platynereis dumerilii* 48 hours post fertilization (hpf). *P. dumerilii*, is an interesting biological model, sometimes considered a "living fossil" as it is a slow-evolving protostome that has been shown to possess ancestral cell types, and thus may provide a better comparison with vertebrates than fast evolving species like *Drosophila* and nematodes where derived features can obscure evolutionary signal [17], [18].

**Figure 2:**
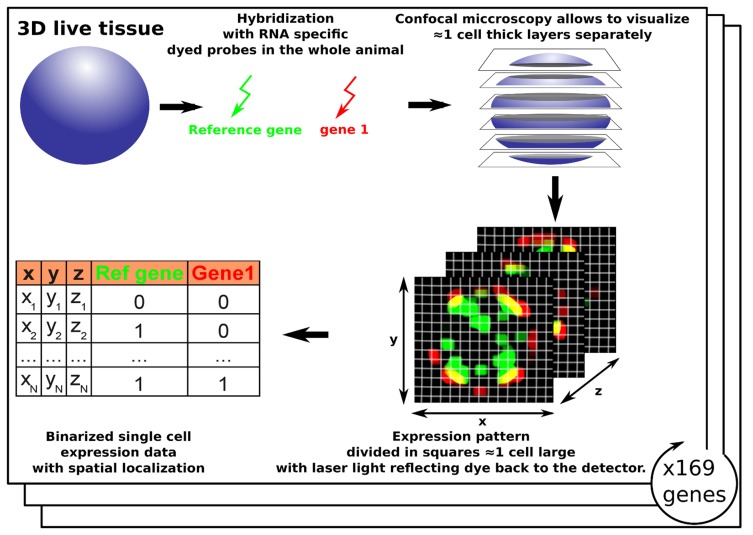
Wholemount in-situ hybridization expression data for 86 genes in the full brain of Platynereis. The whole larvae is hybridized with two dyed probes targetting specific mRNAs, one corresponding to a reference gene and the other a gene of interest. Using confocal microscopy, the whole larvae is visualized slice by slice and the dyed regions are reported with laser light reflecting back to the detector. Every image is then divided into 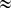
1 cell large squares which allows the reconstruction of the 3D map of expression for the two genes in the full brain. The process was repeated 86 times for key genes in Platynereis development [3].

**Figure 3:**
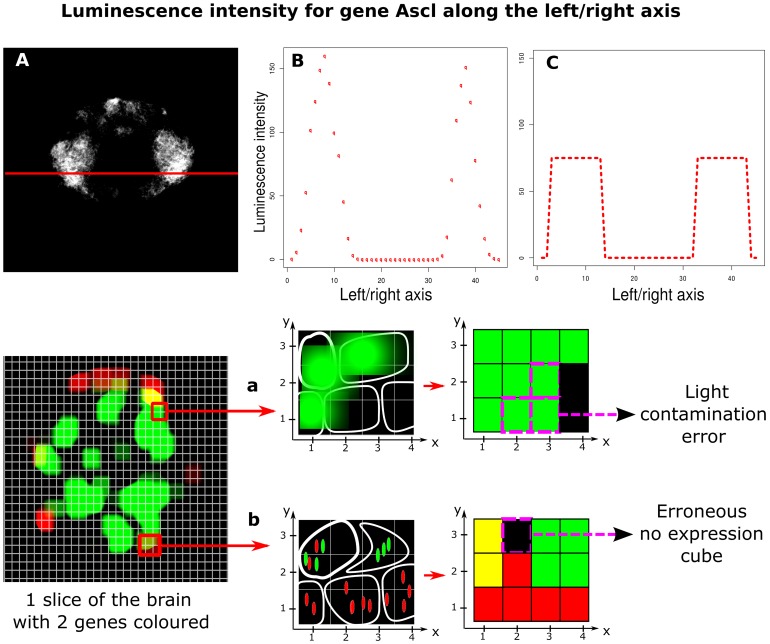
Light contamination in in-situ hybridization luminescence data. Panel A shows the raw fluorescent microscopy capture of the gene Ascl's expression for one layer in the brain of Platynereis. Panel B shows the light intensity measured along the red line in panel A. Panel C shows the expected light intensity profile without light contamination. Because of the small scale of study, voxels surrounded by other voxels expressing a particular gene will have a higher intensity values because of nearby light contamination. Panel D shows errors introduced by the voxel cell model. Path *a* shows how regions with highly expressed genes can introduce errors through light contamination. Path *b* shows how some voxels may appear artificially void of expression because of the uneven distribution of transcripts inside the cytoplasm especially for large cells.

Wholemount in-Situ hybridization (WiSH) is an experimental technique where the practitioner uses labelled probes designed to be specific to a given mRNA to determine in which cells of the tissue under study that message is expressed. For a small organism like *Platynereis*, the staining can be applied to the whole animal and a 3-Dimensional representation of the expression pattern of a gene can be deduced using confocal microscopy to study the patterns of gene expression slice by slice. In practice, following the staining, imaging and alignment, the brain volume was partitioned into 32,203 3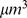 voxels. The 3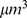 volume was chosen to be slightly smaller than the average cell in *Platynereis*'s brain but it is possible to consider this grid as a simple cellular model where each voxel roughly corresponds to a cell in the brain. Within each voxel, the light emission (assumed to be correlated to the gene's expression level) was measured (Figure 2). Theoretically, this luminescence data is quantitative but, on such a small scale, light contamination between voxels means that the quantitative measurements have to be interpreted with caution (Figure 3). Additionally, the light efficiency of probes can differ leading to a high experiment-to-experiment variability. Consequently, we binarized the dataset by setting the value of expression within a voxel to 1 or 0, depending upon whether the gene was or was not expressed, respectively (see Discussion).

By repeating this process with different probes, expression patterns for 86 genes of interest were mapped. Importantly, due to the stereotypic nature of early *Platynereis* development [17], the expression patterns can be overlaid, meaning that for each 3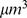 voxel it is possible to determine which subset of the 86 genes is expressed. We can represent this information in a 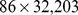 matrix of binary gene expression, where the location of each voxel, roughly representing a cell within the brain, is referenced in a 3D coordinate system. Given this coordinate system, we can create a neighbouring graph representation, where each node in the equivalent undirected graph corresponds to a voxel in the in-situ data. The edges of the graph were computed following a simple neighbouring system taking only the 6 closest neighbours, one in each direction of the 3D space.

### Clustering method

Markov random fields (MRF) are statistical models that provide a way of modeling entities composed of multiple discrete sites such as images where each site is a pixel or, in our case, a biological tissue where each site is a single voxel roughly corresponding to a cell, in a context-dependent manner [19]. MRF based methods find their roots in the field of statistical mechanics as the Ising model [14] and its generalization, the Potts model [15]. Since then, they have been and are still mainly used in the field of image analysis, and the literature about them is ever growing [20]–[22]. More specifically, MRF methods are found in a wide range of applications such as image restoration and segmentation [23], surface reconstruction [24], edge detection [25], texture analysis [26], optical flow [27], active contours [28], deformable templates [29], data fusion [30] and perceptual grouping [31]. MRFs have also been used in a variety of biological applications from analysing medical imaging data [23], [32], [33] to analysing networks of genomic data [34]. Additionally, the Cellular Potts Model [35] has been used to model tissue development at a sub-cellular resolution.

Mathematically, MRF models are built around two complementary sub-models. The field represents the sites and their spatial structure. The Hammersley-Clifford (1971) theorem states that the probability distribution of the Markov field can be represented as a Gibbs measure, which incorporates an energy function into which the spatial coherency parameters of the model are incorporated. Some critical choices in terms of the modeling framework are the structure of the neighbourhood system and the energy function. The emission model is used to describe the underlying data (gene expression measurements in our case) and it is necessary to make some assumptions about its form depending upon the underlying data.

In our study the goal is to allocate the 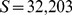 voxels described above into 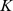 clusters, where 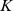 is unknown, using the binarised matrix of 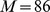 gene expression measurements, 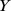. To incorporate spatial information into our clustering scheme, we assume that 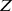, the (latent) vector of length 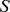 that describes the allocation of voxels to clusters, satisfies a first-order Markov Random Field (MRF), where the probability that a voxel is allocated to a given state depends only upon the states of its immediate neighbours. Additionally, within cluster 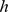 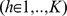, we assume that the expression of gene 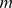 follows a Bernoulli distribution with parameter 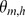; we denote the full set of Bernoulli parameters using the 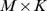 matrix 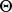. In a typical MRF, the degree of spatial cohesion is determined by a single parameter 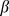, which is assumed to be constant for all clusters [36], [37]. However, in the context of tissue organisation, it is reasonable to expect that the degree of spatial cohesion will differ between clusters; consequently, we estimate a separate value of 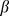 for each of the 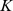 clusters (Methods). To estimate the parameters we use a fully-factorized variational expectation maximization (EM) approach in conjunction with mean-field approximations to infer intractable values [16]. To choose the optimal number of clusters, 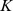, we use the Bayesian Information Criterion (BIC).

### Model validation and comparison with alternative approaches

**Figure 4:**
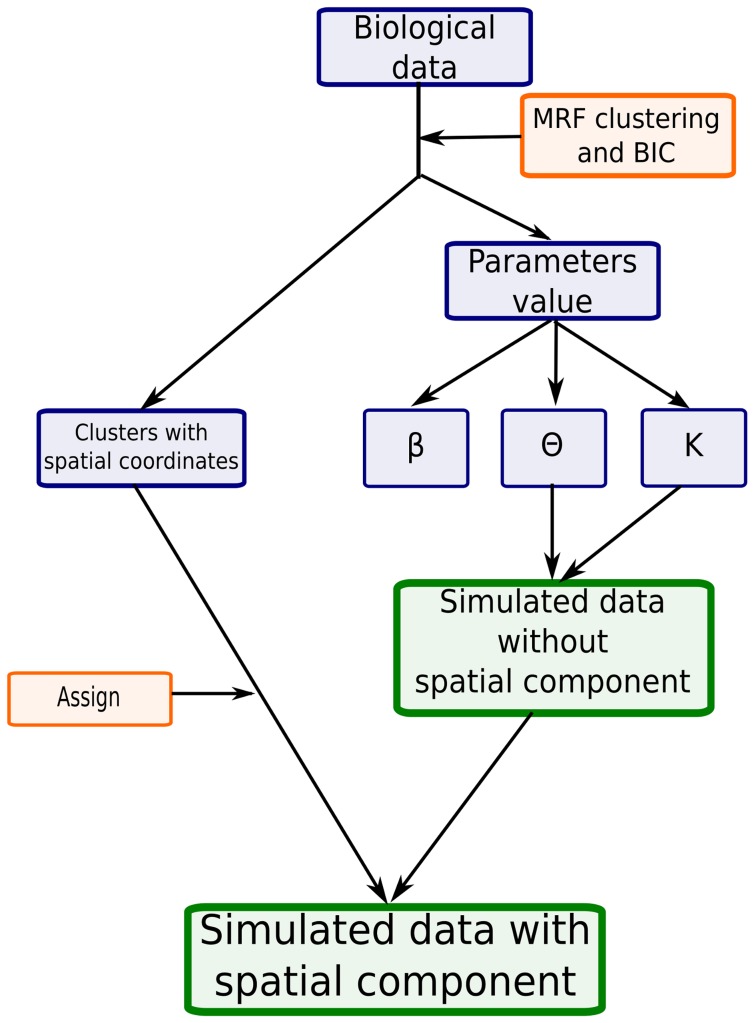
Simulation scheme used to generate gene expression data with a spatial component and known parameters. The values of 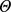 are used to generate a dataset of clusters with the same gene expression profile as the reference. Each simulated voxel is then assigned to its corresponding spatial localization so that the simulated data keeps the spatial component of the biological data.

Simulating data with a spatial component is a non-trivial problem. Existing methods rely on MCMC approaches as described in [38]. However, in our case with a relatively large number of nodes in the graph (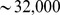), this is computationally expensive. To overcome this problem, we exploited the fact that the *Platynereis* dataset already possesses a spatial structure, and use this as a synthetic example on which to base our simulations. As outlined in Figure 4, we start by clustering the gene expression data using different values of 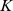 and store the corresponding parameter estimates. Subsequently, the estimated Bernoulli parameters, 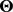, were used to simulate binarised gene expression data from 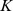 clusters where, for each voxel contained within cluster 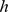, the expression of gene 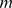 is simulated from a Bernoulli distribution with parameter 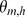 (Figure 4).

Next, each simulated voxel was assigned to the same spatial location as the corresponding voxel in the biological dataset. As a result, the simulated and the biological datasets have the same neighbouring graph. We can then cluster these simulated datasets using the method outlined above and determine how accurately we can estimate the parameters (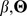) and choose the correct number of clusters, 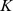.

The most important criterion for assessing the efficacy of our approach is the similarity between the inferred and true clusters. This also implicitly assesses the accuracy of the estimation of 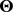: if the inferred and true clusters are identical, the estimates of 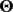 must be equal to the true values. In practice, we used the Jaccard coefficient to compare the inferred and the true clusters (Methods), where a Jaccard coefficient of 1 implies perfect agreement. To benchmark our approach's performance, we also assessed the ability of two other models to cluster the simulated data: hierarchical clustering (hClust), a very widely used approach in genomics and elsewhere, and an independent mixture model, which allows the relative improvement in performance added by the spatial component to be studied.

Additionally, the likelihood function that needs to be maximised possesses many stationary points of different natures. Thus, convergence to the global maximum with the Expectation-maximisation algorithm (see Methods section), depends strongly on the parameter initialisation. To overcome this problem, different initialisation strategies have been proposed and investigated (see for instance [39]–[41]). Herein, we compare a random initialisation scheme with an initialisation based upon the solution obtained by applying hClust.

**Figure 5:**
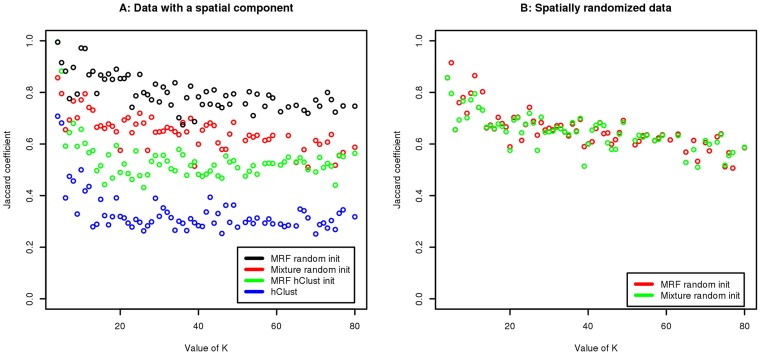
Jaccard coefficient between “true” and resulting clusters on the simulated data with different methods and initializations. Panel A compares the performance of the MRF method with a randomly initialization with an independent mixture model also with a random initialization, the MRF method initialized with the hClust classification and hClust alone on data simulated with a spatial component. Panel B shows the Jaccard coefficient for the MRF method and independent mixture model both with a random initialization; in this case both methods are applied to simulated data that lacks a spatial component.

The results of these experiments are shown in Figure 5 for 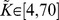. Our method, when used with a random initialization scheme (Methods), has an average Jaccard coefficient of 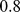, and clearly demonstrates better performance than the other methods. The second best performing method is the independent mixture model with a random initialization, which has an average Jaccard coefficient of 0.7. Since the independent mixture approach is equivalent to the MRF with all the 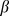 parameters set equal to 0 (i.e., without a spatial component) this suggests that accounting for the spatial aspect yields improved results. Given this, it is perhaps unsurprising that hClust also performs relatively poorly. Additionally, we note that initializing the MRF with the hClust output yields results that are superior to those generated by hClust but that are still poorer than either the randomly initialized independent mixture model or the MRF approach. This is likely explained by noting that, depending upon the initialization, the EM algorithm might converge to a local maximum. Consequently, for the rest of this study we use the random initialization strategy to initialize the EM algorithm.

**Figure 6:**
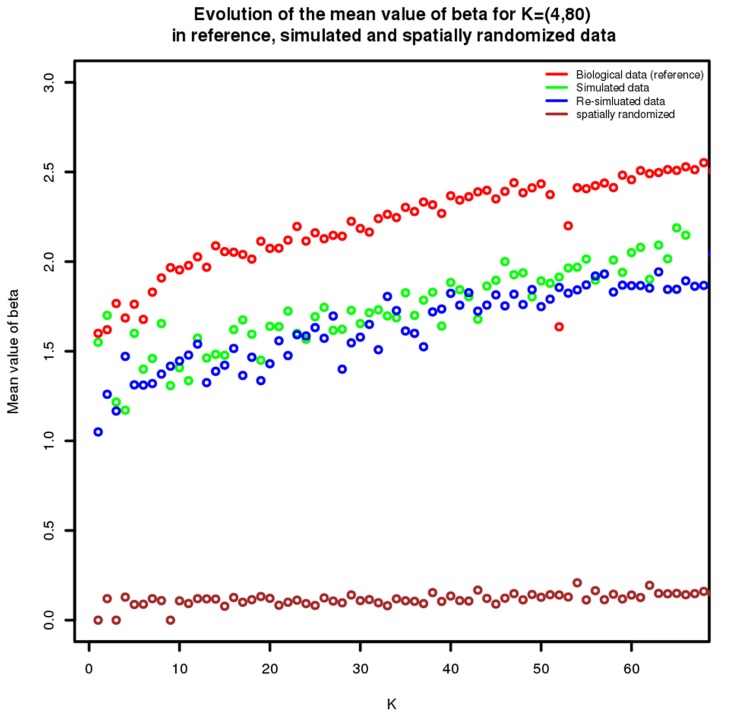
Validating the estimation of beta. This figure shows the evolution for 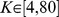 of the mean value of 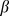 across all the clusters. The red dots represent the biological data clustering (i.e the reference in our simulations scheme). The green dots represent the results obtained after clustering simulated data, which shows an underestimation of 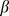. To confirm that this underestimation come from the simulation scheme and not the clustering method, we used the simulated data as the reference to generate a "second generation" of simulated data, suppressing the simulation scheme bias (see Figure 7). The results of this re-simulation are shown by the blue dots, which exhibit no underestimation of 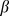. Finally the brown dots represent the mean value of 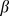 on the same simulated data but spatially randomized, as expected the 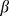 are now estimated to 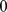.

**Figure 7:**
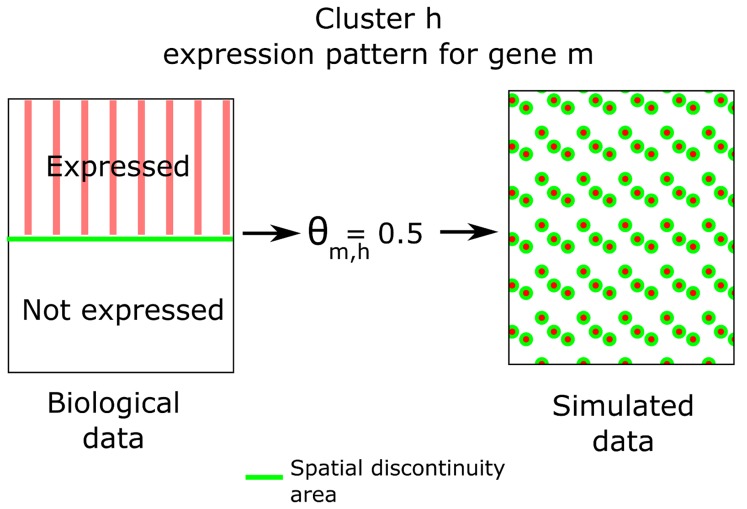
Decrease in spatial coherency due to the simulation scheme. For an example cluster 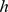, gene 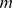 may only be expressed in half of the voxels. This will yield 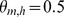. However, in the biological data, the voxels expressing gene 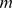 may be spatially coherent (i.e., located close to one another), leading to a reduced area of expression discontinuity (the green line). By contrast, in the simulated data the expression of such a gene will lose its spatial coherency, leading to an increased area of expression discontinuity. The number of voxels having a neighbour with some differences in the gene expression pattern is directly linked to the value of 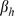 through the energy function (Methods). This explains the underestimation of 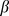 observed in Figure 6.

As well as directly comparing the clusters, we can also determine how accurately the 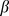 parameters are estimated. To this end, in Figure 6 we compare the true and inferred mean values of 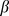 for different values of 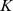. The values of 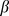 increase with 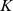, which is to be expected since more clusters implies the existence of more transition areas, thus making an increase of 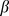 necessary to maintain the optimal spatial coherency of the model. Figure 6 also shows a slight but consistent underestimation of 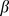. This can be explained by noting that the simulation scheme used may reduce the spatial coherency within clusters. Specifically, as illustrated in Figure 7, clusters may not display homogeneous expression of a given gene: instead, depending upon the value of 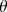, a gene will be expressed only in a fraction of voxels. In reality, the voxels in which such genes are expressed may have a coherent spatial structure within the cluster that is lost in the simulation, thus explaining the consistently smaller values for 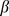 that are estimated. To confirm this, we performed a second simulation using the parameter values estimated from the first simulation as a reference. In this context we did not expect any further loss of spatial coherency, which was indeed confirmed as shown by the blue curve in Figure 6.

To validate further our estimation of 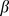, we randomized the coordinates of the voxels to lose any spatial component before re-clustering the data. As expected, we observed that the estimates of 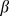 were very close to 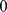 for all clusters (Figure 6), as well as there being very similar Jaccard coefficient values (relative to the true values) for the independent mixture and the MRF model. Both of these observations provide confidence in our assertion that the spatial component plays an important role in the fit.

**Figure 8:**
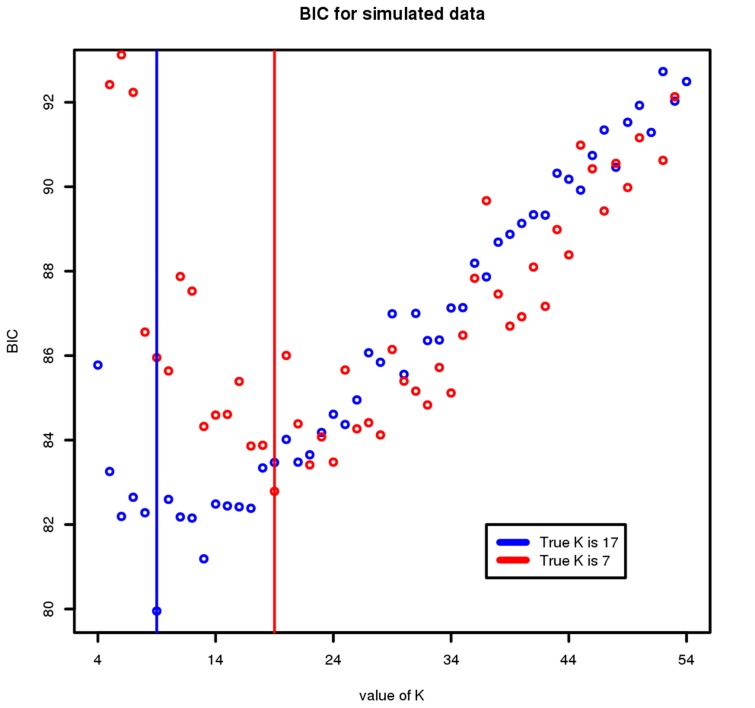
Estimating the BIC from the simulated data. The BIC is plotted on the y-axis for different values of K on the x-axis. The red and the grey points correspond to the BIC estimated when the underlying data have 17 and 7 clusters, respectively. The minimum BIC value is 18 and 7, respectively, suggesting that the MRF approach in conjunction with the BIC well estimates the optimal number of clusters.

Finally, we assessed the ability of the model to choose the correct number of clusters, 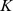. To do this, we noted the "true" number of clusters underlying the simulated data and compared this with the chosen value, 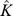. The results for two representative choices of 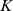 are shown in Figure 8 and demonstrate that our clustering approach, in conjunction with the BIC, is able to accurately determine the optimal number of clusters.

### Biological interpretation

After validating our method using simulated data, we next studied the biological meaning of each of the 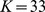 clusters generated by applying the HMRF model to the real data. To do this, we combined each cluster's spatial location with its corresponding expression parameter 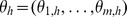. The latter parameter allows a stereotypical expression "fingerprint" to be associated with every cluster.

In practice, not all of the 86 genes will provide insight into the biological function of a given cluster. For instance, in the case of a ubiquitously expressed gene, 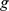, the value of 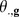 will be high for all clusters. To overcome this problem, we developed a score, 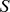, for each gene, 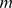 and each cluster 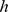, where:



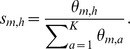



For each gene, 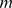, and cluster, 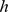, 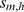 is large if gene 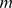 is specific to cluster 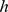. Consequently, the top scoring 3 or 4 genes for each cluster will represent a specific stereotypical expression pattern that will help us infer or confirm the identity of the functional tissue represented by each cluster.

**Figure 9:**
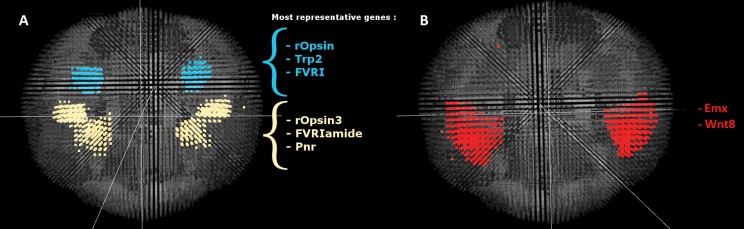
Eyes and mushroom bodies in the brain of Platynereis as clustered by the HMRF method. Panel A: Adult and larval eyes in separate clusters with their top 3 most representative genes. Panel B: Mushroom bodies and their most representative genes. This visualization has been captured using the software bioWeb3D [53].

**Figure 10:**
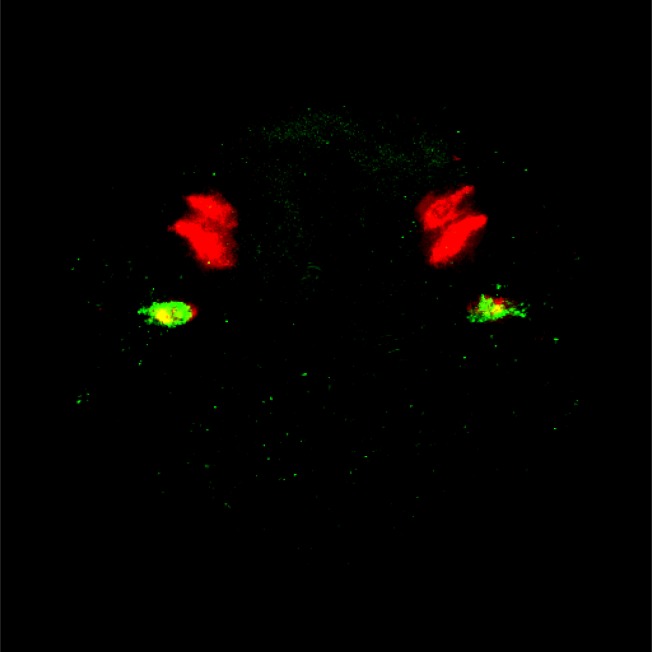
In-situ hybridization image for rOpsin and rOpsin3 in the full brain at 48hpf (Apical view). Z-projection of the expression of rOpsin (red) in both the adult eyes and the larval eyes, rOpsin3 (green) specifically in the larval eyes and co-expression areas in some areas of the larval eyes in the full brain of *Platynereis* at 48hpf. This image been obtained directly from the data obtained in [3]

To provide confidence in our approach, we first considered well characterised regions within the *Platynereis* brain. Arguably the best-studied regions of the brain in *Platynereis* are the eyes: the brain has 4 eyes, two larval and two adult, and their locations and expression fingerprints are well known. As shown in Figure 9A, our approach generates two spatially coherent clusters that correspond to each of these regions. Importantly, the genes that best characterise these clusters are biologically meaningful: *rOpsin* and *rOpsin3*, both members of the well-described opsin family of photosensitive molecules [42], [43], best distinguish the adult eye and larval eyes respectively, consistent with the in-situ data images shown in Figure 10. As well as the eyes, a second region of the *Platynereis* brain, the mushroom bodies (which corresponds to the pallium, layers of neurons that cover the upper surface of the cerebrum in vertebrates [3]), are also clearly identified by our approach (Figure 9B).

**Figure 11:**
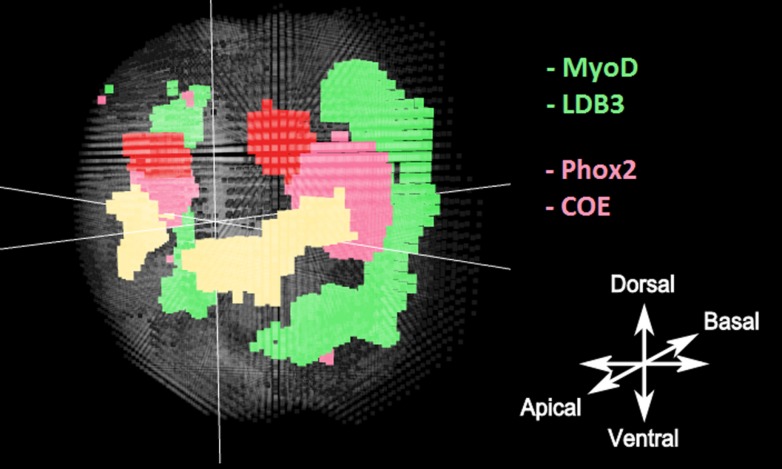
A putative tissue of developing neurons between the eyes and the larvae's developing muscles. The yellow and red clusters are the eyes as seen on figure 9. The green cluster represents the developing muscles on the basal side of the larvae, as the location and the most specific genes strongly suggest. The pink cluster is a putative tissue that makes an interesting link between the eyes and the muscles. The most representative gene of this tissue is Phox2, a homeodomain protein required for the generation of visceral motor-neurons in *Drosophila* [49]

As well as identifying clusters corresponding to known cell types, we also identified clusters that might correspond to less well studied subtypes with specific biological functions. In Figure 11, the green cluster defines a region on the basal side of the larvae that can be associated both by its localization and by its most representative genes (*MyoD* [44], [45] and *LDB3* [46], [47]) with the starting point of the developing muscles of the adult animal. Indeed, *MyoD* has been shown to play a key role in the differentiation of muscles during development in vertebrates and invertebrates [44], [45] and *LDB3* codes for the protein LDB3, which interacts with the myozenin gene family that has been implicated in muscle development in vertebrates [47].

Given the location of the eyes and the developing muscles, the location of the pink cluster in Figure 11 is interesting. This cluster surrounds the larval eyes, the adult eyes and reaches the hypothetically developing muscles described above. Looking at the most representative genes for this pink cluster, it is interesting to note the presence of *Phox2*, a homeodomain protein that has been shown to be necessary for the generation of visceral motor-neurons (neurons of the central nervous system that project their axons to directly or indirectly control muscles) as described generally in [48] and in *Drosophila* [49]. The second most representative gene, *COE*, has also been shown to play a role in *Platynereis* and *Drosophila* neural tissue development [50]. In this context, although we lack biological validation, we can hypothesise that the cells within this particular cluster could be developing neurons that link the eyes to the muscles of *Platynereis*. Although this hypothesis remains purely speculative and would need validation in the laboratory, we believe this example is an interesting proof-of-concept that our clustering method can prove useful for hypothesis generation. Indeed, the analysis of the parameter values and the spatial localization attached to the clusters has allowed us to place with a reasonable level of confidence a functional hypothesis about a tissue that was not clearly defined either spatially or functionally. It is also interesting to note that hClust does not separate either putative region when clustering the same data with the same number of clusters.

**Figure 12:**
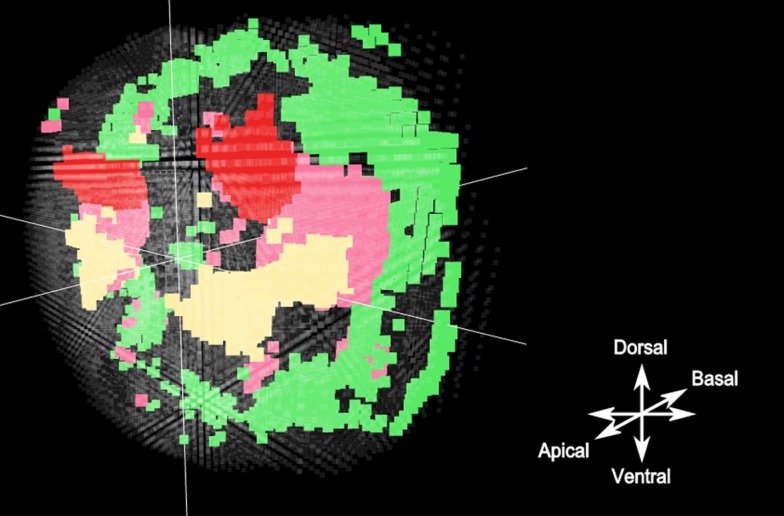
Clusters obtained with the independent mixture model. The Adult eyes are well isolated (red). The larval eyes (yellow) as well. The muscles and the region of potential developing neurons are picked up as well. However if all the regions are recognizable, they are extremely poorly defined spatially with a lot of noise and little spatial coherency. This noise leads to biologically incoherent regions to be clustered with known cell types, particularly for the potential pool of developing neurones as well as the developing muscles.

When we used an independent mixture model approach (i.e., with no spatial component) to cluster the data the results were more comparable to those obtained when using the HMRF strategy. However, as can be observed when comparing Figures 12 and 11, the clusters generated via the independent EM approach are considerably noisier and, as expected, less spatially coherent than those generated by the HMRF model. Further, for the developing muscle region, this noise is linked to biological imprecisions. When compared to in situ data generated by Fischer *et al*. [17], who used a phalloidin in situ stain to investigate the location of the muscles at this developmental stage, it can be observed that the muscles are restricted to regions located away from the axes of symmetry, more consistent with the HMRF clustering output. Similarly, the independent mixture model method associates to the hypothesized region of developing neurons around the eyes, some ventral areas that seem unlikely to belong to the same sub tissue. Consequently, it seems likely that the HMRF not only performs better than the independent mixture model on simulated data but also better reflects the underlying biology.

## Discussion

### Data binarization

**Figure 13:**
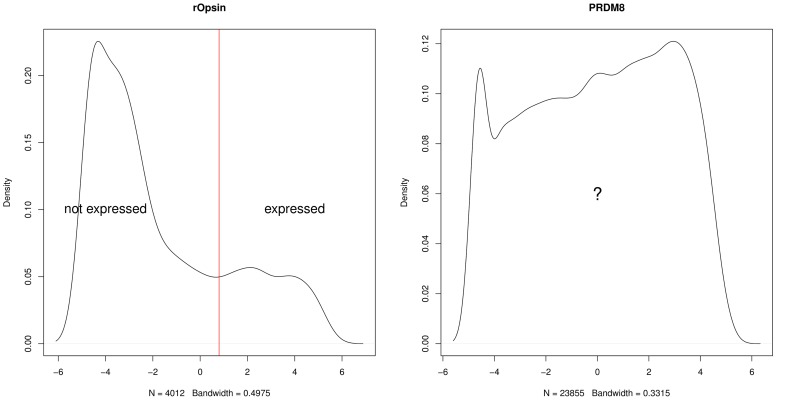
Densities of log luminescence values for two genes (rOpsin, PRDM8) over the 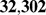 voxels. For *rOpsin*, the density exhibits two clear peaks making the choice of a binarizing threshold easy. By contrast, for *PRDM8* there is no such clear threshold, making an automated binarization method hard to implement.

As shown in Figure 3, we overcame problems linked to light contamination by binarizing the "quantitative" luminiscence information. To do this, it is necessary to specify a threshold above which a gene is considered expressed. Ideally the same threshold would be applied to all genes — however, when we examined the density plots of light intensities for each gene we observed significant differences that rendered such an approach impossible. Specifically, for some genes, the density of intensities clearly separated the voxels into two groups, corresponding to those where the gene is expressed and unexpressed, respectively (Figure 13 (left)). For the remaining genes, however, the density plot was diffuse, with no clear separation of the voxels into expressed and unexpressed clusters (Figure 13 (right)). Consequently, we binarized each gene manually by choosing an optimal threshold based upon inspection of the raw fluorescent microscopy images. This is possible since the number of genes under study is relatively small. However, as the number of genes for which data is available increases (as will be the case, for example, with single-cell RNA-sequencing studies), an automated method, perhaps based upon mixtures of Gaussians in the context of the WiSH data, will be required.

Importantly, if the noise level in single cell expression datasets decreases to the extent where we can safely consider the results as quantitative, our method can easily be transformed to take this feature into account. The general outline of the model will stay exactly the same, the change will occur in the emission distribution. Instead of representing a Bernoulli parameter for each gene and each cluster, each 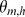 could instead represent the parameter of a Poisson distribution.

### Validity of the model's independence hypothesis

In our model we assume that, conditional upon the allocation of a voxels to a cluster, the gene expression levels can be described by independent Bernoulli distributions. This is a reasonable assumption in the context of the 86 genes chosen by Tomer et al. [3], since they were selected to have largely orthogonal expression profiles. In other words, they were chosen since they were known to correspond to distinct and potentially interesting regions of the *Platynereis* brain. However, in many other settings a larger number of genes, many with correlated expression profiles (i.e., genes in the same regulatory network) will be profiled and this assumption will be invalid. Consequently, extending the model to allow for dependence structure in the emission distributions will be a critical challenge. Additionally, as the number of genes increases, our approach for choosing the most specific genes will become less practical. Instead, entropy based approaches, such as the Kullback-Leibler divergence, might be more suitable.

### Summary

In summary, we have illustrated, using both simulations and real data, that accounting for spatial information significantly improves our ability to cluster voxels roughly representing brain cells into coherent and biologically relevant sets. While our approach converges very quickly (on the order of minutes) for the motivating dataset described herein, as the volume of data increases (i.e., by assaying the expression levels of thousands of genes in each cell using single-cell RNA-sequencing) it will be important to carefully investigate how easily our model scales. Nevertheless, we anticipate that our method will play an important part in facilitating interpretation of single-cell resolution data, which will be an increasingly important challenge over the next few years.

## Methods

In this section we describe the Hidden Markov Random Field based approach that we developed to cluster the in-situ hybridization data into 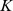 clusters (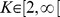). Subsequently, we will describe our approach for choosing 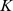.

Let 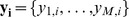 be the gene expression measurement for each voxel 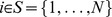 where 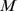 is the number of considered genes. Originally, 169 gene expression patterns where generated, but due to experimental constraints — confocal laser microscopy artefacts and high background noise in some samples — we filtered out 83 of those genes to create a gold standard dataset with 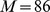 manually validated genes. Our goal is to assign each voxel, 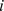, to one of the 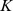 possible clusters. We define a set of discrete random variables 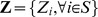 that represents the cluster each voxel is assigned to. Each 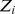 takes a value in 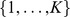 denoting the 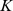 possible clusters. The aim of the method is to restore the unknown clustering structure with regard to gene expression similarity as well as spatial dependencies between voxels. To do this, we assume that the 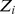 are dependent variables and we encode the spatial relationship using a neighbourhood system defined through a graph 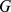.

In this work, we use a first order neighbourhood system, i.e, the 6 closest sites. The set of voxels is then represented as a graph 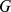 with edges projecting from each voxel to its closest neighbours. The dependencies between neighbouring voxels are modelled by assuming that the joint distribution of 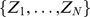 is a discrete **MRF**:



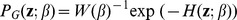
where 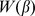 is a normalising constant summed over all the possible configurations 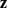 that soon becomes intractable as the number of sites increases. 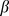 is a set of parameters, and 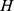 is the energy of the field. This energy can be written as a sum over all the possible cliques of the graph 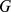. We restrict this summation to the pairwise interactions and the function 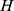 is assumed to be of the following form:



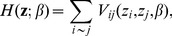
(1)where 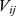 represents the pair-wise potentials, i.e the dependency between 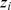 and 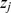 for two neighbouring voxels 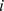 and 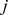. The Potts model [15] traditionally used for image segmentation, is the most appropriate discrete random field of the form of (1) for clustering as it tends to allocate neighbours to the same cluster, thus increasing the spatial coherency. The Potts model is defined by the Energy function 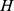:



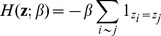
with 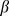 the interaction parameter between two neighbours. Note that the greater the value of 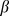, the more weight is given to the interaction graph (i.e., there is more spatial cohesion). Although this feature is appealing for clustering, the standard Potts model penalizes the interaction strength in different clusters with the same penalty. In practice, given the nature and the biological context of our data, it may be more appropriate to allow cell types that are more spatially coherent to have a higher value of 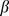 (i.e stronger interaction) than other cell types, in other words to use adaptive smoothness related to the type of cells in the cluster. To this end, we propose a variant of the Potts model, which we define as:



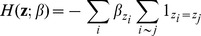



This extended version allows each cluster 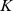 to have its own parameter for interaction strength. For the model to be fully defined, we need to specify, besides the prior described above for the labels 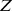, the emission model. To this end, a Bernoulli distribution is used as the sampling distribution:



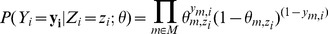



The 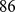 genes selected can be considered as independent because they are all key genes in the development of the brain of *P. dumerilii*. Consequently, we can assume conditional independence of the observed variables 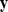 given the clustering 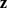. This leads to:



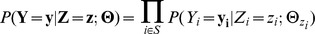



The log likelihood of the complete model is thus given by:



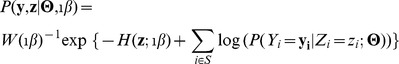



We denote the parameters of the model as 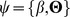.

As mentioned before, our aim is to assign each voxel 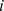 to one of the 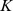 possible clusters. To do so, we chose to consider the Maximum Posterior Marginal (MPM) that maximizes 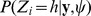, where the 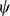 are unknown and need to be estimated. We solved this problem using the EM algorithm [51]. For HMRFs, contrary to independent mixture models, the exact EM can not be applied directly due to the dependence structure and some approximations are required [13]. We chose to use approximations based on the Mean field principle [16]. We used this to approximate the posterior probabilities 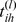 that voxel 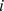 belongs to cluster 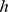 at iteration 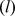. We also used a mean-field approximation to approximate the value of the intractable normalizing constant 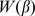 (details are given in the Appendix). Once the 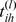
's are computed, we assign each voxel to the cluster 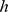 for which this posterior probability is the highest.

After the E step, maximizing 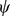 is relatively straightforward. For 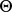, once the the 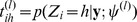 have been computed during the E-step, we use those probabilities to assign each voxel to its cluster at iteration 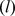. Once the new partition is created, the maximization of 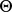 can be computed for cluster 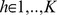 and gene 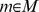 with 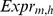 the number of voxels expressing gene 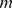 in cluster 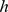 and 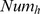 the total number of voxels in cluster 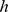.



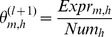



To maximize 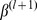, we iteratively applied a gradient ascent algorithm, the positive version of the gradient descent algorithm [22] to the function 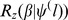 for each 
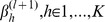


A detailed description of the algorithm is described in Text S1.

### Choosing K

**Figure 14:**
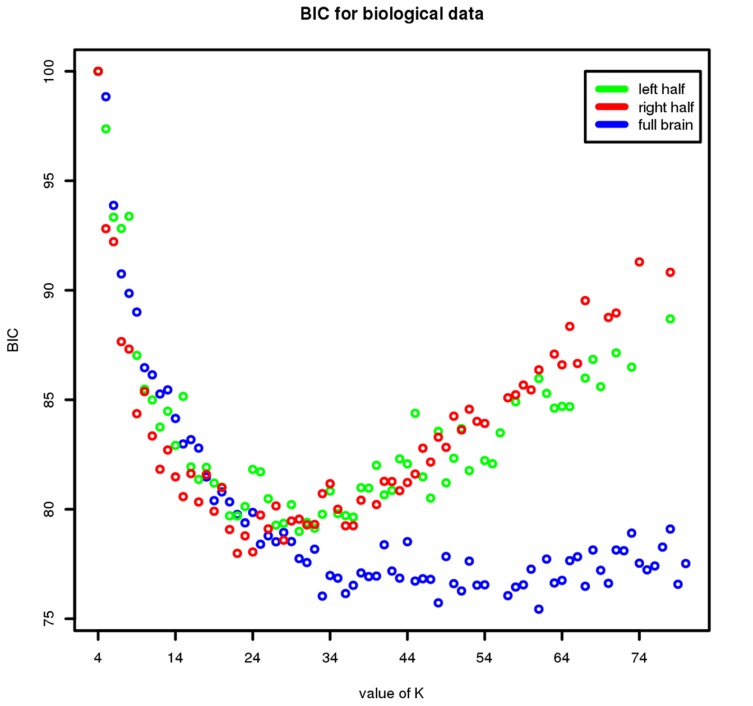
BIC results on biological data. Results are shown for 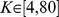 (x axis) with the full brain, and the two left and right half separately. The y axis shows the BIC value in % of the highest BIC value for each dataset.

To select the optimal number of clusters we used the BIC [45], which finds the optimal number of clusters, 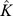, by selecting the value of 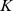 that minimises its value. However, due to the symmetry of the brain we used a slightly different approach. As shown in Figure 14 (blue dots), the BIC does not reach a clear minimum when applied to all voxels in the brain but instead reaches a plateau after a given number of clusters. This is most likely due to the highly, but not perfectly symmetrical nature of the brain: with a small 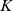, the same "tissue" on both the left and the right hand side of the brain will belong to the same cluster. However, because the two sides of the brain are not perfectly symmetrical, as 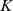 increases the left and right part of the same "tissue" will be clustered separately. As a result, the likelihood continues to increase sufficiently to explain the flattened BIC curve. Moreover, this hypothesis seems to be confirmed by the fact that when computing the BIC on the right and left side of the brain separately, the curve has in both cases a clear minimum as shown in Figure 14 (red and green dots). Given this, we opted to choose 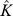 as the point where the BIC curve reaches a plateau.

### Data and code availability

The data are available as a binarized datset of single cell gene expression data for the 86 genes in the brain of *Platynereis dumerilii*. An implementation of the EM algorithm in the C programming language is also available on the Github page of the project [52].

## Supporting Information

Text S1 **Mean field approximations and EM procedure.** We provide more information about the mean field approximation used to estimate both the conditional probabilities of a voxel belonging to a particular cluster given the parameter values as well as the intractable normalizing constant. We also present pseudo-code to outline the EM Mean-field algorithm used in our HMRF implementation.
(PDF)

## References

[1] Montagna W, Parakkal P (1974) Structure and function of skin. Elsevier.

[2] Elowitz MB, Levine AJ, Siggia ED, Swain PS (2002) Stochastic gene expression in a single cell. Science 297: 1183–1186. 12183631 10.1126/science.1070919

[3] Tomer R, Denes AS, Tessmar-Raible K, Arendt D (2010) Profiling by image registration reveals common origin of annelid mushroom bodies and vertebrate pallium. Cell 142: 800–809. 20813265 10.1016/j.cell.2010.07.043

[4] Perou CM, Jeffrey SS, Van De Rijn M, Rees CA, Eisen MB, et al (1999) Distinctive gene expression patterns in human mammary epithelial cells and breast cancers. Proceedings of the National Academy of Sciences USA 96: 9212–9217. 10.1073/pnas.96.16.9212 PMC17759 10430922

[5] Tautz D, Pfeifle C (1989) A non-radioactive in situ hybridization method for the localization of specific RNAs in drosophila embryos reveals translational control of the segmentation gene hunchback. Chromosoma 98: 81–85. 2476281 10.1007/BF00291041

[6] Tang F, Barbacioru C, Wang Y, Nordman E, Lee C, et al (2009) mRNA-Seq wholetranscriptome analysis of a single cell. Nature Methods 6: 377–382. 19349980 10.1038/nmeth.1315

[7] Shapiro E, Biezuner T, Linnarsson S (2013) Single-cell sequencing-based technologies will revolutionize whole-organism science. Nature Reviews Genetics 14: 618–630. 10.1038/nrg3542 23897237

[8] Eisen MB, Spellman PT, Brown PO, Botstein D (1998) Cluster analysis and display of genome-wide expression patterns. Proceedings of the National Academy of Sciences USA 95: 14863–14868. 10.1073/pnas.95.25.14863 PMC24541 9843981

[9] Tavazoie S, Hughes JD, Campbell MJ, Cho RJ, Church GM (1999) Systematic determination of genetic network architecture. Nature Genetics 22: 281–285. 10391217 10.1038/10343

[10] Tamayo P, Slonim D, Mesirov J, Zhu Q, Kitareewan S, et al (1999) Interpreting patterns of gene expression with self-organizing maps: methods and application to hematopoietic differentiation. Proceedings of the National Academy of Sciences USA 96: 2907–2912. 10.1073/pnas.96.6.2907 PMC15868 10077610

[11] Lein ES, Hawrylycz MJ, Ao N, Ayres M, Bensinger A, et al (2007) Genome-wide atlas of gene expression in the adult mouse brain. Nature 445: 168–176. 17151600 10.1038/nature05453

[12] Eberwine J, Sul JY, Bartfai T, Kim J (2014) The promise of single-cell sequencing. Nature Methods 11: 25–27. 24524134 10.1038/nmeth.2769

[13] Dang M, Govaert G (1998) Spatial fuzzy clustering using EM and markov random fields. International Journal of System Research and Information Science 8: 183–202.

[14] Ising E (1925) Beitrag zur Theorie des Ferromagnetismus. Zeitschrift für Physik A Hadrons and Nuclei 31: 253–258.

[15] Wu FY (1982) The Potts model. Reviews of modern physics 54: 235.

[16] Celeux G, Forbes F, Peyrard N (2003) EM procedures using mean field-like approximations for Markov model-based image segmentation. Pattern recognition 36: 131–144.

[17] Fischer A, Henrich T, Arendt D (2010) The normal development of Platynereis dumerilii (Nereididae, Annelida). Frontiers in zoology 7: 31. 21192805 10.1186/1742-9994-7-31 PMC3027123

[18] Arendt D (2005) Platynereis dumerilii: a living fossil elucidating the evolution of genomes and of the CNS. Theory in Biosciences 124: 185–197.17046355

[19] Li SZ, Singh S (2009) Markov random field modeling in image analysis, volume 3. Springer.

[20] Rozanov YA (1982) Markov random fields. Springer.

[21] Li H, Kallergi M, Clarke L, Jain V, Clark R (1995) Markov random field for tumor detection in digital mammography. IEEE Transactions on Medical Imaging 14: 565–576. 18215861 10.1109/42.414622

[22] Bishop CM, et al. (2006) Pattern recognition and machine learning, volume 1. New York: Springer.

[23] Zhang Y, Brady M, Smith S (2001) Segmentation of brain MR images through a hidden Markov random field model and the expectation-maximization algorithm. IEEE Transactions on Medical Imaging 20: 45–57. 11293691 10.1109/42.906424

[24] Paulsen RR, Baerentzen JA, Larsen R (2010) Markov random field surface reconstruction. IEEE Transactions on Visualization and Computer Graphics 16: 636–646. 20467061 10.1109/TVCG.2009.208

[25] Zerubia J, Chellappa R (1993) Mean field annealing using compound Gauss-Markov random fields for edge detection and image estimation. IEEE Transactions on Neural Networks 4: 703–709. 18267770 10.1109/72.238324

[26] Clausi DA, Yue B (2004) Comparing cooccurrence probabilities and Markov random fields for texture analysis of SAR sea ice imagery. IEEE Transactions on Geoscience and Remote Sensing 42: 215–228.

[27] Heitz F, Bouthemy P (1993) Multimodal estimation of discontinuous optical flow using Markov random fields. IEEE Transactions on Pattern Analysis and Machine Intelligence 15: 1217–1232.

[28] Martın-Fernández M, Alberola Lopez C (2005) An approach for contour detection of human kidneys from ultrasound images using Markov random fields and active contours. Medical Image Analysis 9: 1–23. 15581809 10.1016/j.media.2004.05.001

[29] Mignotte M, Meunier J, Tardif JC (2001) Endocardial boundary e timation and tracking in echocardiographic images using deformable template and markov random fields. Pattern Analysis & Applications 4: 256–271.

[30] Wright W (1989) A Markov random field approach to data fusion and colour segmentation. Image and vision computing 7: 144–150.

[31] Fields SUMR (1997) Perceptual grouping of contour segments using markov random fields. Pattern Recognition and Image Analysis 7: 11–17.

[32] Held K, Kops ER, Krause BJ, Wells W, Kikinis R, et al (1997) Markov random field segmentation of brain MR images. IEEE Transactions on Medical Imaging 16: 878–886. 9533587 10.1109/42.650883

[33] Descombes X, Kruggel F, von Cramon DY (1998) Spatio-temporal fMRI analysis using Markov random fields. IEEE Transactions on Medical Imaging 17: 1028–1039. 10048860 10.1109/42.746636

[34] Wei Z, Li H (2007) A Markov random field model for network-based analysis of genomic data. Bioinformatics 23: 1537–1544. 17483504 10.1093/bioinformatics/btm129

[35] Voss-Bohme A (2012) Multi-Scale Modeling in Morphogenesis: A Critical Analaysis of the Cellular Potts Model. PLoS One 7: e42852. 22984409 10.1371/journal.pone.0042852 PMC3439478

[36] Subudhi BN, Bovolo F, Ghosh A, Bruzzone L (2014) Spatio-contextual fuzzy clustering with markov random field model for change detection in remotely sensed images. Optics & Laser Technology 57: 284–292.

[37] Zhang H, Shi W, Wang Y, Hao M, Miao Z (2014) Spatial-attraction-based Markov random field approach for classification of high spatial resolution multispectral imagery. IEEE Geoscience and Remote Sensing Letters 11: 489–493.

[38] Chalmond B (1989) An iterative Gibbsian technique for reconstruction of m-ary images. Pattern recognition 22: 747–761.

[39] Biernacki C, Celeux G, Govaert G (2003) Choosing starting values for the EM algorithm for getting the highest likelihood in multivariate gaussian mixture models. Computational Statistics & Data Analysis 41: 561–575.

[40] Karlis D, Xekalaki E (2003) Choosing initial values for the EM algorithm for finite mixtures. Computational Statistics & Data Analysis 41: 577–590.

[41] McLachlan G, Peel D (2004) Finite mixture models. John Wiley & Sons.

[42] Terakita A (2005) The opsins. Genome Biology 6: 213. 15774036 10.1186/gb-2005-6-3-213 PMC1088937

[43] Randel N, Bezares-Calderón LA, Gühmann M, Shahidi R, Jékely G (2013) Expression dynamics and protein localization of rhabdomeric opsins in platynereis larvae. Integrative and comparative biology 53: 7–16. 23667045 10.1093/icb/ict046 PMC3687135

[44] Weintraub H, Davis R, Tapscott S, Thayer M, Krause M, et al (1991) The myoD gene family: nodal point during specification of the muscle cell lineage. Science 251: 761–766. 1846704 10.1126/science.1846704

[45] Michelson AM, Abmayr SM, Bate M, Arias AM, Maniatis T (1990) Expression of a MyoD family member prefigures muscle pattern in Drosophila embryos. Genes & Development 4: 2086–2097. 2176634 10.1101/gad.4.12a.2086

[46] Krcmery J, Camarata T, Kulisz A, Simon HG (2010) Nucleocytoplasmic functions of the PDZ-LIM protein family: new insights into organ development. Bioessays 32: 100–108. 20091751 10.1002/bies.200900148 PMC3010972

[47] Marziliano N, Mannarino S, Nespoli L, Diegoli M, Pasotti M, et al (2007) Barth syndrome associated with compound hemizygosity and heterozygosity of the TAZ and LDB3 genes. American Journal of Medical Genetics Part A 143: 907–915. 17394203 10.1002/ajmg.a.31653

[48] Brunet J, Pattyn A (2002) Phox2 genes-from patterning to connectivity. Current Opinion in Genetics & Development 12: 435. 12100889 10.1016/s0959-437x(02)00322-2

[49] Briscoe J, Sussel L, Serup P, Hartigan-O'Connor D, Jessell T, et al (1999) Homeobox gene Nkx2. 2 and specification of neuronal identity by graded Sonic hedgehog signalling. Nature 398: 622–627. 10217145 10.1038/19315

[50] Demilly A, Simionato E, Ohayon D, Kerner P, Garcès A, et al (2011) Coe genes are expressed in differentiating neurons in the central nervous system of protostomes. PLoS One 6: e21213. 21695052 10.1371/journal.pone.0021213 PMC3117877

[51] Dempster AP, Laird NM, Rubin DB, et al (1977) Maximum likelihood from incomplete data via the EM algorithm. Journal of the Royal Statistical Society 39: 1–38.

[52] Project source on github. URL https://github.com/jbogp/MRF_Platynereis_2014.

[53] Pettit JB, Marioni JC (2013) bioWeb3D: an online webGL 3D data visualisation tool. BMC Bioinformatics 14: 185. 23758781 10.1186/1471-2105-14-185 PMC3710502

