# Supplementary material for: Identifying Cell Types from Spatially Referenced Single-Cell Expression Datasets
Source: PLoS Comput Biol. 2014 Sep 25;10(9):e1003824. doi: 10.1371/journal.pcbi.1003824 (PMC4177667; doi:10.1371/journal.pcbi.1003824)
Supplement: Text S1 — Mean field approximations and EM procedure. We provide more information about the mean field approximation used to estimate both the conditional probabilities of a voxel belonging to a particular cluster given the parameter values as well as the intractable normalizing constant. We also present pseudo-code to outline the EM Mean-field algorithm used in our HMRF implementation. (PDF) [file pcbi.1003824.s001.pdf]

Appendix

Mean field approximations

Below we provide more information about the mean field approximation used to estimate both the conditional probabilities of a site (cell) belonging to a particular cluster given the parameter values as well as the intractable normalizing constant.

First, at iteration (l), we define the model's full expectation as:

$$Q(\boldsymbol{\psi} \mid \boldsymbol{\psi}^{(l)}) = \underbrace{\sum_{\boldsymbol{z}} p(\boldsymbol{z} \mid \boldsymbol{y}; \boldsymbol{\psi}^{(l)}) \log\ p(\boldsymbol{y} \mid \boldsymbol{z}; \Theta)}_{R_y(\Theta \mid \boldsymbol{\psi}^{l})} + \underbrace{\sum_{\boldsymbol{z}} p(\boldsymbol{z} \mid \boldsymbol{y}; \boldsymbol{\psi}^{(l)}) \log\ p(\boldsymbol{z} \mid \boldsymbol{\beta})}_{R_z(\boldsymbol{\beta} \mid \boldsymbol{\psi}^{(l)})}$$

Subsequently, if z_i denotes the cluster that site i is allocated to, we can re-write R_y as:

$$R_y(\Theta \mid \boldsymbol{\psi}^{(l)}) = \sum_{i \in S} \sum_{z_i=1}^{K} [\log\ f_{z_i}(\boldsymbol{y_i}; \Theta)]\ p(Z_i = z_i \mid \boldsymbol{y}; \boldsymbol{\psi}^{(l)})$$

Here, $f_{z_i}(z_i \in K, i \in S)$ denotes the emission density associated with cluster z_i, such that:

$$\begin{aligned} f_{z_i}(\boldsymbol{y_i} \mid z_i; \Theta) &= f_{z_i}(\boldsymbol{y_i} \mid z_i; \boldsymbol{\theta_{z_i}}) \\ &= \prod_{m \in M} \theta_{m,z_i}^{y_{m,i}} \times (1 - \theta_{m,z_i}^{1-y_{m,i}}) \end{aligned}$$

Given this, we can define the intractable probability, $t_{i\,h}^{(l+1)}$, that a site, i belongs to cluster h at iteration $(l+1)$ as:

$$t_{i\,h}^{(l+1)} = p(Z_i = h \mid \boldsymbol{y}; \boldsymbol{\psi}^{(l)})$$

The mean field approximation allows us to write the following fixed point equation:

$$t_{i\,h}^{l+1} \approx \frac{f_h(y_i; \boldsymbol{\theta}_h^{(l)})\ exp\{\beta_h^{(l)}\ \sum_{j \in N(i)} t_{j\,h}^{(l+1)}\}}{\sum_{u=1}^{K}\ f_u(y_i; \boldsymbol{\theta}_u^{(l)})\ exp\{\beta_u^{(l)}\ \sum_{j \in N(i)} t_{j\,u}^{(l+1)}\}}$$

Similarly, it can be noted that R_y contains an intractable normalizing constant, $W(\boldsymbol{\beta})$, which can be factorized using the mean field approximation as:

$$W(\boldsymbol{\beta}) = \sum_{\boldsymbol{z'}} exp(-H(\boldsymbol{z'})) \approx \sum_{i \in S} \sum_{\boldsymbol{z_i}} exp(-H(\boldsymbol{z_i})) = \sum_{i \in S} \sum_{\boldsymbol{z_i}} exp(\beta_{z_i} \sum_{j \in N(i)} 1[z_i = z_j])$$

EM algorithm

Below we use pseudo-code to outline the EM Mean-field algorithm used in our HMRF implementation.

Listing 1: EM Mean-field algorithm in pseudo-code

```
/*retrieving parameters*/

/*Starting initialization*/
  /*Reading initialization file*/
  if(initialization provided) {
    Read initialization file
    Assign cluster values to z^(0)
  }
  /*Random initialization*/
  else {
    /*Generating R random initializations*/
    for(R runs) {
      Generate random initialization
      Compute field likelihood (the R_z part of the full likelihood)
    }
    Select initialization with highest likelihood
    Assign cluster values to z^(0)
  }

/*
*z^(0) is now defined
*Compute the initial parameters values
*/
  For every cluster h, gene m, compute:
    θ^(1)_{m,h} = arg max_Θ R_y(Θ | ψ^(0)) = Expr_{m,h} / Num_h
  With Expr_{m,h} the number of sites expressing m in cluster h
  And Num_h the number of sites in cluster h in z^(0)

  Set β^(1) to the user defined value

/*Start EM procedure*/
  /*Alternate E step and M step until convergence is reached*/
  while(Clusters changed < user defined convergence limit) {
    /*Iteration l*/
    /*E step*/
    Compute densities f_{z_i}(y_i | z_i; Θ^(l)) from the emission model
    Fixed point algorithm to compute t^(l)_{ih}
    /*Create z^(l)*/
    Assign each site i to its most probable cluster using
    t^(l+1)_{ih} = p(Z_i = h | y; ψ^(l))

    /*M step*/
    Compute Θ^(l+1) from z^(l)
    Gradient ascent algorithm to compute β^(l+1) using the approximate
     form of W(β)
  }

/*Output clustering results*/
```
